# Medial Trap Door Technique to Harvest a Cancellous Bone Graft From the Anterior Iliac Crest

**DOI:** 10.7759/cureus.48111

**Published:** 2023-11-01

**Authors:** Satheesh G, Vijaykumar Jain S, Chinnaiah R, Nalinkumar S, Hemnaath R

**Affiliations:** 1 Oral and Maxillofacial Surgery, Adhiparasakthi Dental College and Hospital, Melmaruvathoor, IND

**Keywords:** anterior iliac crest, cancellous bone graft, alveolar bone grafting, trap door technique, iliac bone graft

## Abstract

Alveolar bone grafting (ABG) in cleft lip and palate patients allows for the facilitation of eruption of the canine and sometimes eruption of the lateral incisor. It provides bony support to the cleft raising the alar base of the nose and also facilitates the closure of an oro-nasal fistula. Many report at a time when late alveolar bone grafting is the only option to overcome the bony defect mainly due to their socioeconomic concern. Autologous bone graft for alveolar bone grafting harvested from the anterior iliac crest using the medial trap door technique has many advantages over other techniques of harvesting bone graft from the anterior iliac crest. In this case report we have discussed a case of bone graft harvesting from the anterior iliac crest using the medial trap door technique for late secondary alveolar bone grafting.

## Introduction

The objectives of alveolar bone grafting (ABG) in cleft alveolus are to provide stabilization of the maxillary arch; facilitation of closure of oro-nasal fistula; facilitate eruption of canine and lateral incisor; provide bony support for the teeth near the cleft region; raise alar base of the nose; and to achieve good periodontal support for the teeth near the cleft region [1]. Initial attempts in treating alveolar cleft defects date back to 1901, when Von Eiselberg used a pedicled bone graft to fill an alveolar defect [2]. Drachter (1914) used tibial bone and periosteum for the closure of a cleft in the alveolus [3]. Between the 1950s and 1960s many cleft surgeons practiced primary and early secondary bone grafting. Since 1964 lot of publications suggest serious growth disturbances can occur to the middle third facial skeleton if bone grafting is done at the early stage. Secondary alveolar bone grafting that is done during the mixed dentition period gained popularity and became well-established procedure to avoid the sequalae of growth disturbance associated with early bone grafting [1,4]. Late secondary ABG is done after the eruption of the canine tooth and has a lower success rate. The best time for doing ABG is when a thinner shell of the alveolar bone covers the erupting canine or lateral incisor tooth closer to the cleft region [1].

Various donor sites have been tried in harvesting cancellous or cortico-cancellous bone grafts. Johanson and Rockert (1961) have proved in their studies that cancellous autogenous bone grafts that have been harvested from the iliac crest or tibia were transformed to the same as the surrounding palatal bone structure [5]. Cranial bone and rib bone have been used before but tooth eruption through these grafts was not demonstrated to be successful as with iliac and tibial bone [6]. Bone graft harvested from the iliac crest is known to be associated with complications, with frequency ranging between 2% and 49% [7]. The most common complication due to iliac crest bone graft harvesting was “donor site pain” and poor cosmetic appearance due to depression and scarring in the iliac crest. Techniques to harvest bone from the iliac crest can be classified as window technique, splitting technique, trapdoor technique and trephine extraction technique [8,9]. The trapdoor method is a surgical technique to harvest bone from the iliac crest where a trap door or bone flap is made in the iliac crest from which cancellous bone is harvested and the cortical bone flap is placed back to restore the cortical iliac crest anatomy [10,11]. In this case report a novel method, the “medial trap door technique” to harvest cancellous bone graft from the anterior iliac crest, is discussed.

## Case presentation

**Figure 1:**
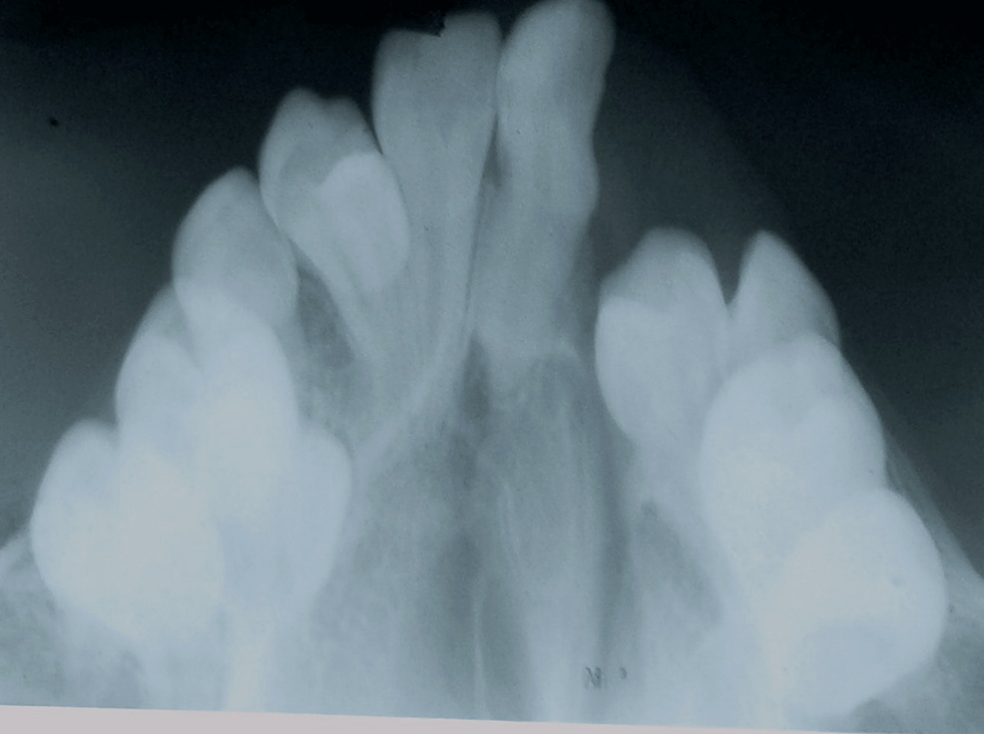
Occlusal radiograph showing cleft region in the alveolus

**Figure 2:**
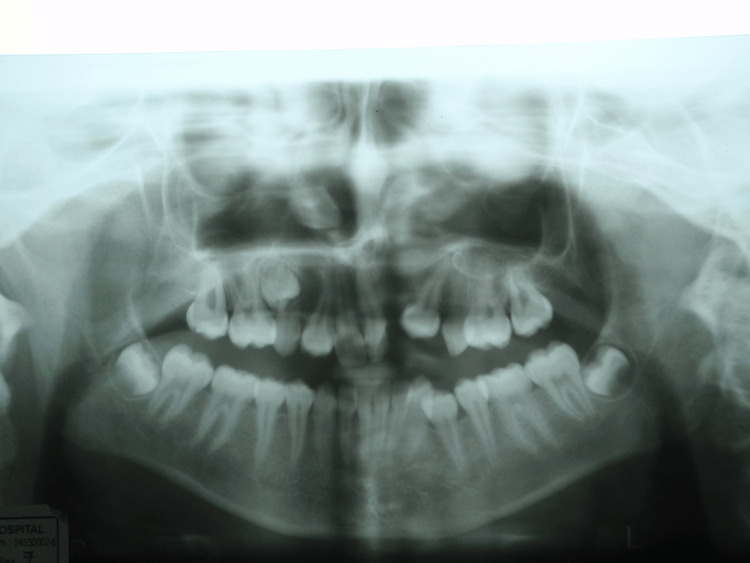
Orthopantomagram (OPG) reveals cleft in the alveolus with impacted 23.

A 17-year-old female patient presented with a cleft in the alveolus on the left side of the upper jaw between 21 and 24 tooth region. An occlusal view radiograph is shown in Figure 1 and an orthopantomagram (OPG) (Figure 2) revealed a bony defect in the alveolus between 21 and 24 tooth region with missing 22 and impacted 23.

**Figure 3:**
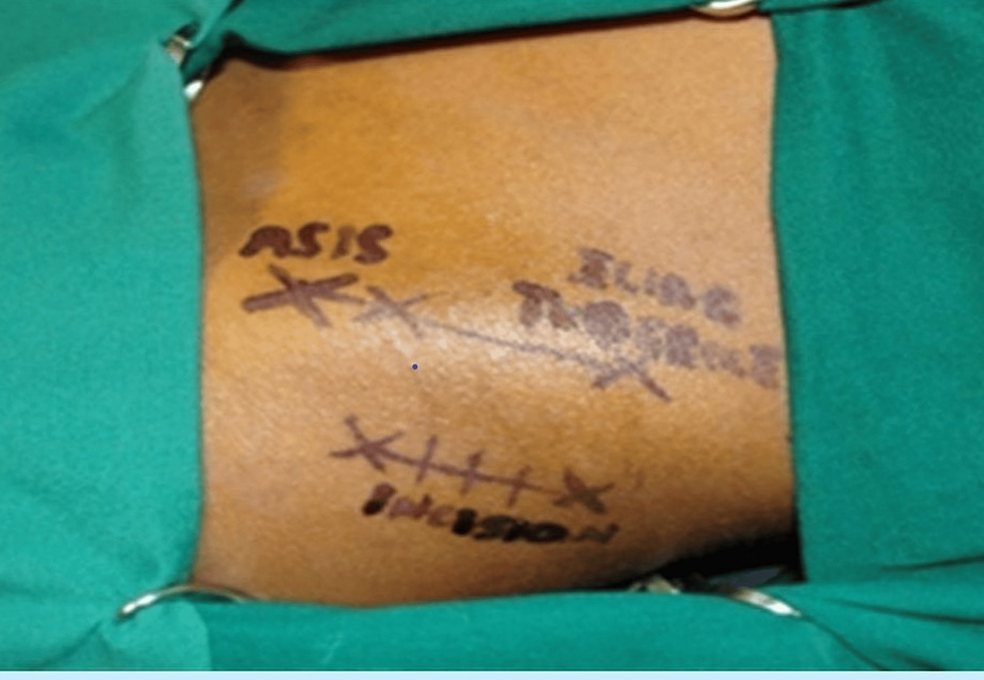
Incision line is marked 2cm lateral to the crest, 2cm in front of the anterior superior iliac spine (ASIS) extending 4cm posterior to the crest

The treatment plan was to do alveolar bone grafting with an autologous cortico-cancellous bone graft from the anterior iliac crest using the medial trap door technique under general anesthesia. The surgical steps that were followed to harvest cortico-cancellous bone from the anterior iliac crest using the medial trap door technique are as follows: the patient is placed in a supine position, and a sandbag is placed beneath the hip region so that it elevates and slightly rotates the anterior iliac crest. Standard methods were followed to prepare the surgical site. The incision line was marked (Figure 3) 2cm lateral to the crest and 2cm in front of the anterior superior iliac spine (ASIS) extending 4cm posterior to the crest

**Figure 4:**
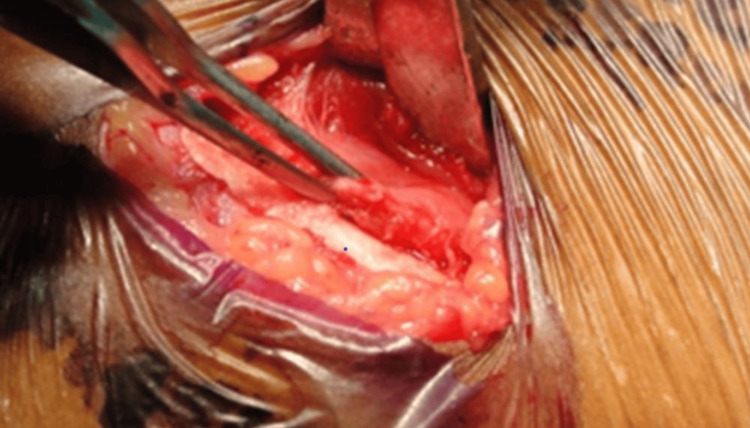
Periosteum is incised staying on the medial aspect of the crest. The periosteum and the iliacus muscle reflected medially

Infiltration of local anesthesia (2% lignocaine with 1:80000 adrenalin) was given over the marked incision line. Skin incision given along the second line by stretching it over the iliac crest then the subcutaneous tissue and superficial fascia incised. The junction between fascia latae and iliacus muscle was medially identified and the junction between fascia latae and iliacus muscle was incised. This exposes the underlying periosteum. Then the periosteum incised staying on the medial aspect of the crest. Periosteum and the iliacus muscle reflected medially as shown in Figure 4.

**Figure 5:**
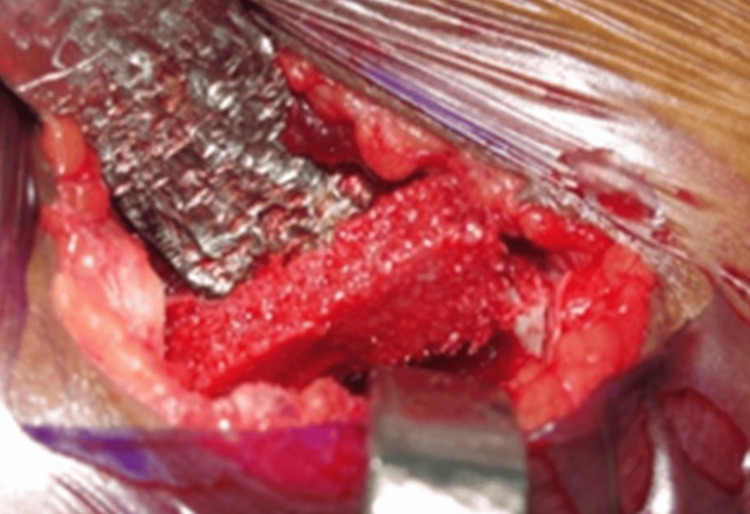
Chunk of cancellous bone harvested

Osteoplastic flap hinged laterally and it provides access to the marrow bone. A chunk of cancellous bone as shown in Figure 5 was harvested.

**Figure 6:**
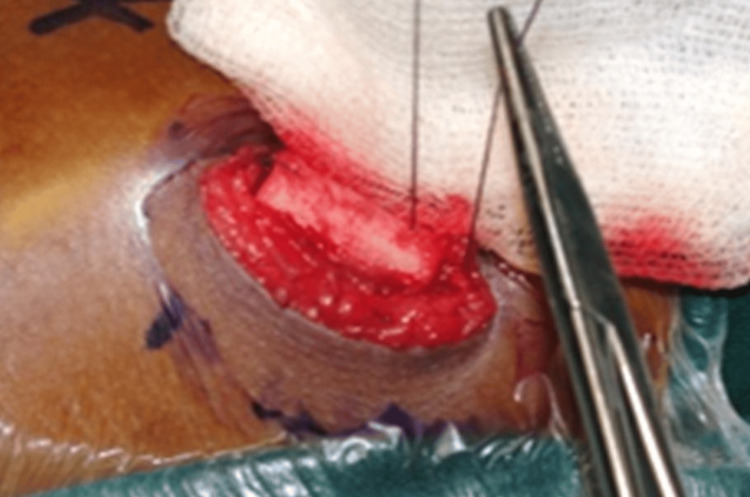
Osteoplastic flap replaced and secured with a strong resorbable suture

**Figure 7:**
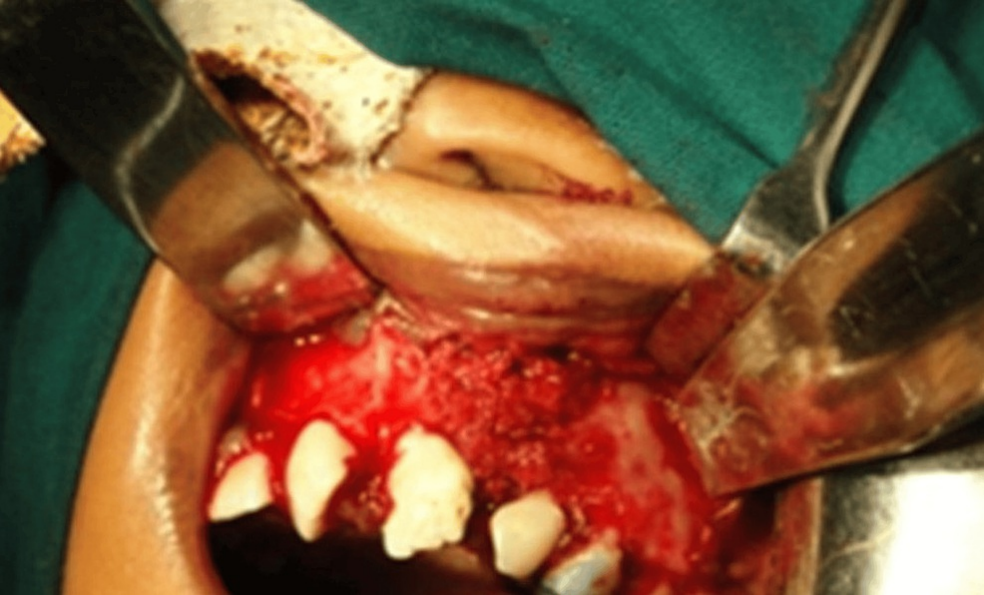
Alveolar bone grafting (ABG) done with the harvested cancellous bone graft

The cortico-periosteal flap is replaced and secured strong with an absorbable suture as illustrated in Figure 6, then the muscle, fascia, and subcutaneous tissue are each closed with 3’0 vicryl, sub cuticular suturing done with 5’0 prolene, ABG done with the harvested cancellous bone graft as illustrated in Figure 7.

To reduce the period of disability and discomfort, early ambulation was advised. Postoperative complications were evaluated weekly at the donor site as pain, infection, paresthesia, hematoma formation, and difficulty in walking (change in gluteal gait), whereas at the recipient site as pain, infection, exposure of graft, rejection of graft, and wound dehiscence. By this technique, sufficient amount of cancellous bone graft was harvested with minimum morbidity to the donor site and ASIS was undisturbed. The postoperative gait of the patient was found to be normal.

## Discussion

Alveolar bone grafting can be primary grafting, secondary grafting or late secondary grafting. Primary grafting is done during the primary dentition period. The primary ABG is seldom used nowadays due to its subsequent bad effects on the growth of the mid-face secondary to encroachment of the vomer-premaxillary suture [1]. Secondary bone grafting is done at the mixed dentition period and the ideal period for ABG will be when a thinner amount of alveolar bone covers the erupting permanent canine [1]. Alveolar bone grafting that is done after the mixed dentition period is termed late secondary ABG and has a low rate of success when compared to early secondary bone grafting. However many patients report late after the eruption of canine for alveolar bone grafting due to varying reasons with socio-economic status being one of them. Autologous iliac bone grafting has better treatment outcomes in terms of graft rejection and osteogenesis when compared with artificial and allogeneic bone grafting [9,10]. In the case presented here, the patient reported for ABG at the age of 17 years. Some advantages of harvesting a bone graft from the anterior iliac crest include larger quantity of marrow bone available to harvest and shorter operative time. Also it has better graft acceptance when compared with artificial and allogeneic bone grafting. On the other hand, some patients had experienced postoperative pain in their iliac bone graft donor sites and developed other complications. The use of the trap door technique successfully decreased some of the complications associated with harvesting bone grafts from the anterior iliac crest, like loss of contour of the iliac crest, injury to lateral cutaneous nerve, gait disturbances, hernia and pain [8,11]. In this case report cancellous bone graft was harvested from the anterior iliac crest using the medial trap door technique in which after harvesting the bone graft osteoplastic flap containing the cortical bone and periosteum was re-approximated and sutured. By this technique injury to the lateral cutaneous nerve can be avoided, loss of contour of the anterior iliac crest can be prevented and also postoperative pain will be minimal and gait disturbance can be avoided.

## Conclusions

In this case report a reliable technique to harvest autologous cancellous bone graft from the anterior iliac crest using the medial trap door technique was discussed. The advantages of this technique include: the chance of gait disturbance was minimal, ASIS undisturbed, chance for perforation of the medial cortex was much less during gouging, donor site morbidity of anterior iliac crest was much minimal, and sufficient amount of cancellous bone can be harvested by medial trap door technique through medial approach. However further studies were needed to substantiate the above advantages of the medial trap door technique to harvest bone grafts from the anterior iliac crest.
